# Transverse Sinus Thrombosis Secondary to Internal Jugular Vein Abnormalities

**DOI:** 10.7759/cureus.15747

**Published:** 2021-06-18

**Authors:** Sherif T Abuserewa, Richard Duff

**Affiliations:** 1 Internal Medicine, Grand Strand Medical Center, Myrtle Beach, USA; 2 Pulmonary and Critical Care Medicine, Grand Strand Medical Center, Myrtle Beach, USA

**Keywords:** internal jugular vein abnormality, transverse sinus thrombosis, general neurology, neuro-surgery, cerebral venous sinus

## Abstract

Cerebral sinus venous thrombosis (CSVT) is a rare disorder that presents with highly variable neurological manifestations ranging from headache, confusion, seizures, coma to stroke-like symptoms. It predominantly affects young adults and children, with female predilection. We are presenting a case of 59-year-old female with left-sided transverse and sigmoid sinus thrombosis most likely secondary to dehydration on top of chronically diminutive left transverse sinus and internal jugular vein.

## Introduction

Cerebral sinus venous thrombosis (CSVT) is a rare disorder with an annual incidence rate of three to four cases per million in adults in developed countries [1]. However, it’s more common in developing countries especially in females during puerperium [2]. It is considered as a variant of stroke representing 0.5%-2% of all types of strokes affecting mainly young adults and children unlike arterial strokes with female predilection reaching up to 75% cases [3-6]. It has multiple etiologies and highly variable clinical manifestations, making it difficult to be diagnosed; however, with the presence of effective medications and advanced neuroimaging, it has become easier to diagnose it and has a favorable prognosis with good neurological outcomes [6].

## Case presentation

A 59-year-old female nonsmoker presented with left-sided headache, disorientation, and confusion starting a few hours before presentation in the emergency department (ED). A week before that, she had abdominal pain, nausea, and multiple episodes of vomiting and diarrhea. A few days later she complained of frequent episodes of moderate left-sided throbbing headaches associated with nausea. In addition, her symptoms were unresponsive to analgesic medications including diphenhydramine and a combination tablet (aspirin/paracetamol/caffeine). She had gone to an outside hospital and had a computed tomography (CT) scan of head without contrast that was negative and then she was sent home. However, she continued to complain of headaches. In the morning on the day of presentation, her left-sided headache became more severe, and she became disoriented, confused, and off-balance. She denied any fever, seizures, loss of consciousness, shortness of breath, chest pain, or palpitations. She also denied any history of head trauma, coagulopathy, or intake of steroids or any contraceptive medications.

**Figure 1:**
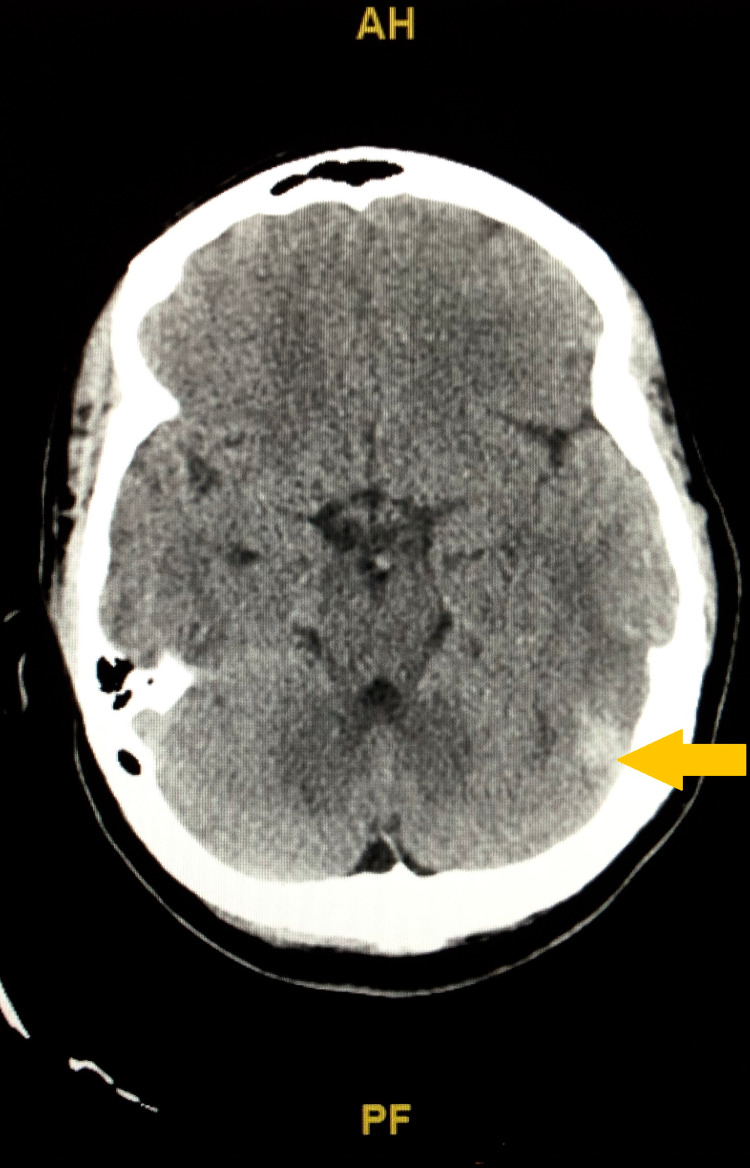
CT of head without contrast showing hyperdensity along the tentorium on the left (yellow arrow) with associated very early hypodensity in the posterior temporal lobe

Her husband drove her to the ED where she was fully conscious, oriented with a Glasgow Coma Scale (GCS) score of 15 and her vitals were stable except for asymptomatic bradycardia. Her neurological examination didn’t show any speech, motor, or sensory deficits and her reflexes were equal bilaterally. She had head CT without contrast showing hyperdensity along the tentorium on the left with associated hypodensity in the posterior temporal lobe on the left as shown in Figure 1, and this raised a concern for small volume infarcts in the posterior left temporal lobe with possible underlying petechial hemorrhage. She had CT angiography (CTA) of the head that showed no evidence of large vessel occlusion, significant intracranial stenosis, aneurysm, or vascular malformation. She was admitted to the ICU.

**Figure 2:**
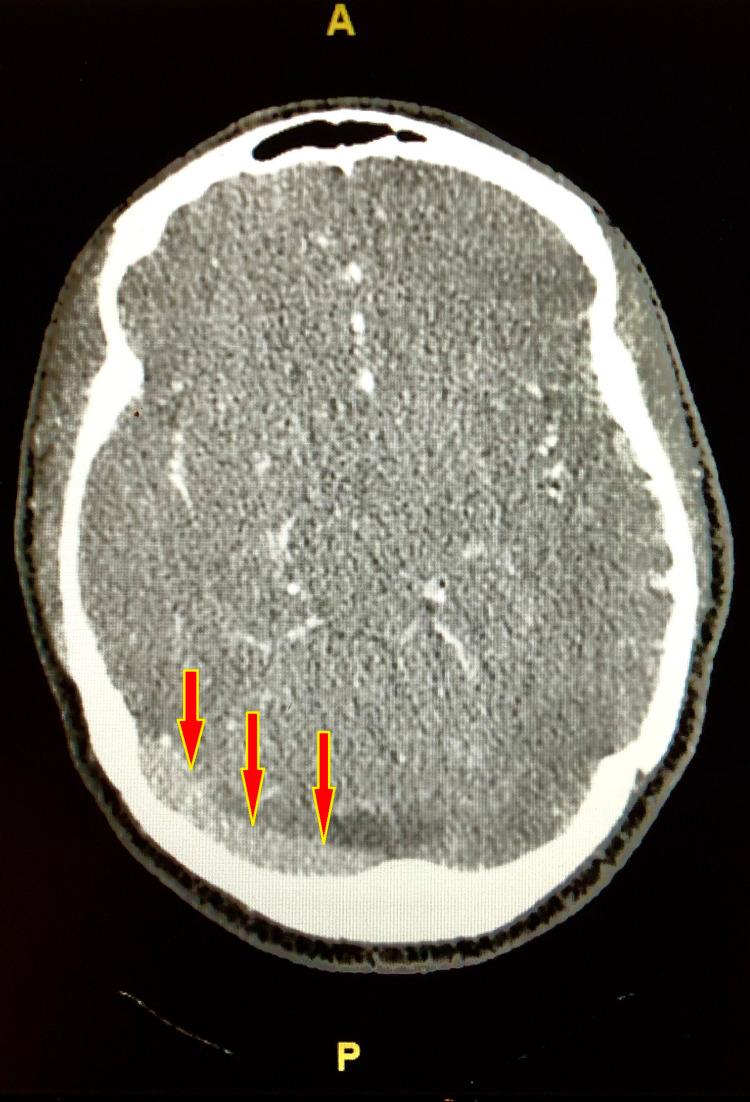
CT venography of head showing widely patent and dominant right cerebral transverse sinus (red arrows) and absence of contrast in the left cerebral transverse or sigmoid sinuses or jugular bulb

**Figure 3:**
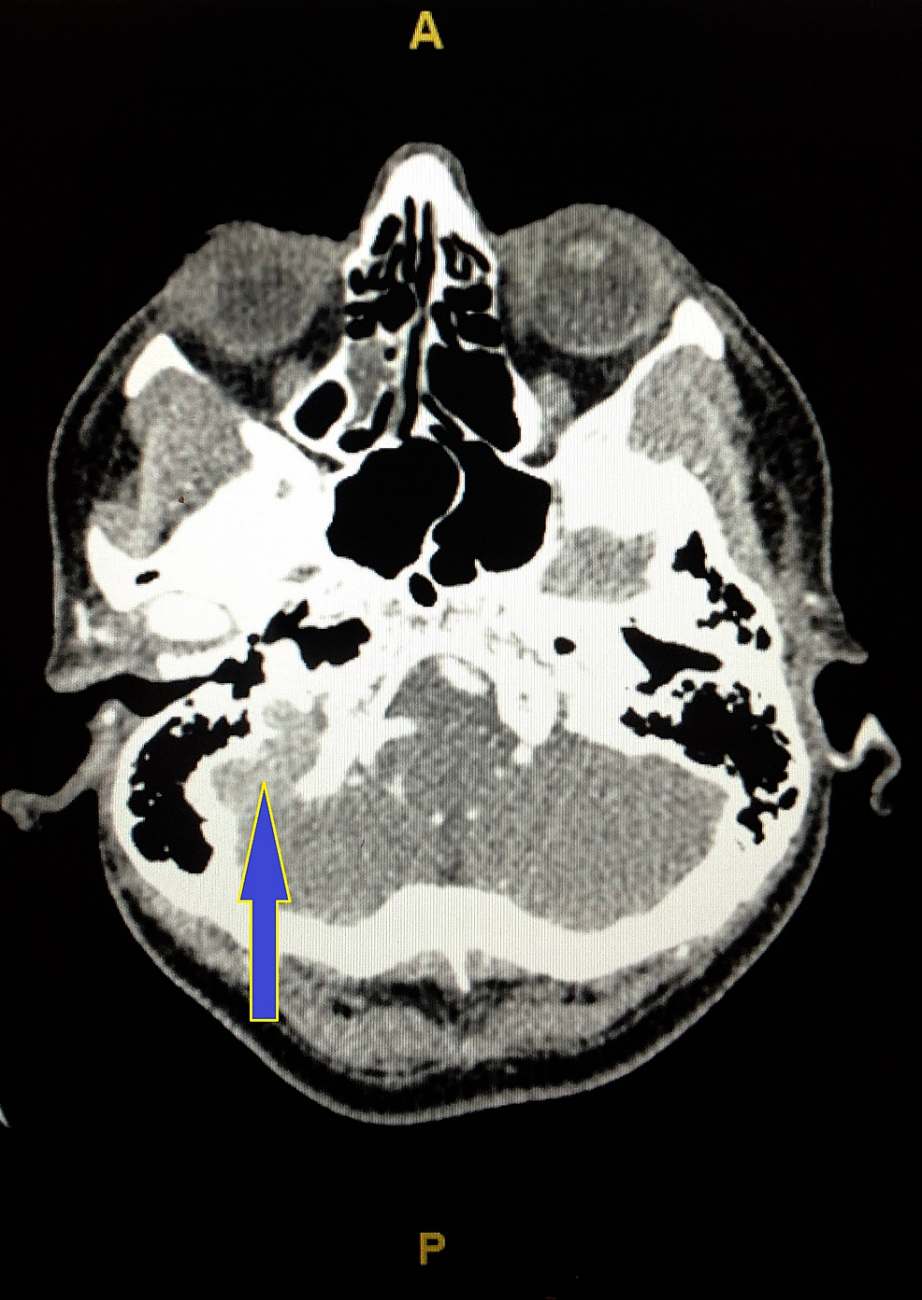
CT venography of head (brain window) showing the contrast in the right sigmoid sinus and internal jugular vein (blue arrow) in the right jugular foramen

**Figure 4:**
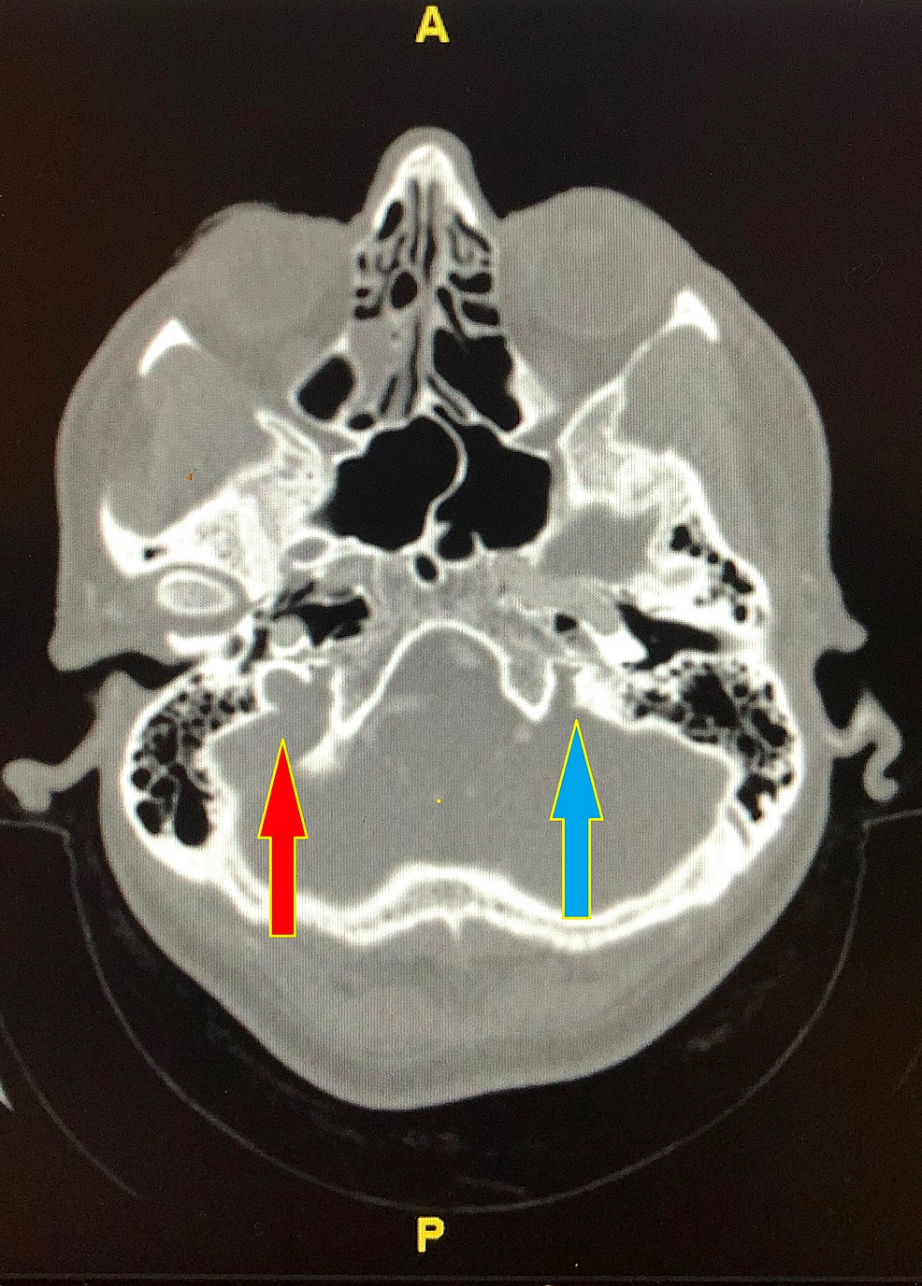
CT venography of head (bone window) showing normal anatomical morphology of the right jugular foramen (red arrow), but on the other side, the left jugular foramen (blue arrow) appears hypoplastic and small in caliber

The neurosurgery team was consulted that recommended CT venography (CTV) of head, which showed widely patent right transverse and sigmoid sinuses, while there was no contrast identified within the transverse or sigmoid sinus (Figure 2). It also showed that the left transverse sinus is likely small in caliber as suggested by the somewhat small in caliber jugular canal (Figures 3, 4).

The patient was started on a heparin drip; the six-hour follow-up head CT hadn’t changed, consistent with dural venous sinus thrombosis and associated developing left temporal lobe infarct. The 24-hour follow-up CT of head without contrast didn’t show any signs of hemorrhage. The patient was then started on warfarin and heparin drip for bridging until therapeutic international normalized ratio (INR) was established. Thrombophilia workup was ordered including lupus anticoagulant, factor V Leiden, factor II activity, protein S, and anticardiolipin Ab that were all within the normal ranges.

The five-week follow-up CTA/CTV of head showed re-canalized but diminutive left transverse sinus with a widely patent right transverse sinus and right sigmoid sinus. Also, it showed diffusely diminutive left internal jugular vein with dominant right internal jugular vein suggesting that the left transverse/sigmoid sinuses were chronically diminutive.

## Discussion

CSVT is a rare condition that predominantly affects young adults and children resulting in venous congestion and brain edema if a cerebral vein is occluded, or an increase in intracranial tension with possible venous congestion complicated with ischemia and infarctions if a venous sinus is occluded, as in this case [5,7].

CSVT’s etiology is multifactorial. It may result from acquired or inherited factors, which meet Virchow’s triad criteria. Inherited thrombophilia is the leading cause of CSVT in developed countries, and it includes homocysteinemia, factor V Leiden homozygous mutation, positive anticardiolipin or anti-phospholipid antibodies, protein C and S and anti-thrombin III deficiency; however in this case, all of them were negative [8,9].

Internal jugular vein abnormalities have a close association with CSVT as these abnormalities may result into insufficient venous drainage leading to venous congestion and CSVT [10]. In this case, CTV showed chronic diminutive left transverse sinus and left internal jugular vein that may be acquired or inherited; however, it was superimposed with dehydration due to her multiple episodes of vomiting and diarrhea that put her at higher risk of thrombophilia. It is less likely that her episodes of vomiting were secondary to increased intracranial tension, as she started to complain of headaches few days after having the episodes of vomiting and diarrhea, and also elevated intracranial tension wouldn’t explain diarrhea. Other acquired risk factors for CSVT include brain tumors, head trauma, central nervous system infections, nephrotic syndrome, and systemic vasculitis [11-15].

Pregnancy, contraceptive medications, and puerperium are also considered high-risk factors associated with CSVT. Studies done before the oral contraceptive era showed no sex predilection, but on the other hand, recent studies have shown that CSVT has female predilection reaching up to 75% cases. Further studies have shown that it is not related to genetic factors but to gender-specific risk factors, mainly oral contraceptives, and to a lesser extent pregnancy and puerperium, where estrogen levels are high leading to a higher prothrombotic effects [16-18].

Iatrogenic causes include lumbar puncture, neurological surgery, and some medications (e.g., cisplatin, methotrexate, steroids) [19,20].

CSVT is presented with variable manifestations and four main patterns, one being the focal pattern, where focal neurological symptoms and signs are present in addition to headache, seizures, or mental status changes. It also can be presented as an isolated intracranial hypertension with its typical manifestations including headache, vomiting and papilledema.

This case was mostly presented with a diffuse subacute encephalopathy pattern where she complained of confusion and disorientation in addition to severe episodic headaches. Sometimes patients are presented with painful eye movements, proptosis, and chemosis secondary to cavernous sinus thrombosis syndrome and this is the fourth clinical pattern of CSVT. Throughout all these different patterns, headache is the most common symptom, reported in 90% of cases. It is usually unilateral and intermittent, which was the typical presentation of this patient [3,21,22].

These patients usually have a head CT scan without contrast as an initial imaging study, to exclude other acute cerebral disorders that could mimic it. It may show hemorrhages or venous infarctions that are the most common findings on CT, found in 40% of cases; however, it may not show any abnormal finding. Although of low sensitivity to CSVT, it may show the thrombus itself inside the blood vessel directly, or may show the effect of venous congestion or ischemia on brain tissue that is referred to as indirect signs [23].

Brain MRI may directly show thrombus in the affected vessel with a higher accuracy than CT, or parenchymal hemorrhages in different brain parts according to the affected sinus. CTV is considered a reliable imaging study with a sensitivity around 95% in comparison to the gold standard digital subtraction angiography; however, in patients with poor kidney functions or at high risk from radiation exposure, magnetic resonance venography (MRV) is preferred. It was noticed that the most sensitive imaging study is the combination of MRI with MRV [24]. Invasive cerebral angiography is the last resort if the diagnosis is not confirmed by the above-mentioned studies. It provides fine details of cerebral veins, and thus can diagnose rare difficult cases.

According to the pathophysiology mentioned above, CSVT may result in increased intracranial tension and/or venous infarctions and hemorrhages, both of which may result into seizures, coma, and death; thus, primary measures of treatment include maintaining patent airways, adequate breathing and oxygenation, elevation of the head of bed, and controlling seizures [25]. Specific measures of treatment are targeting the thrombotic event by anticoagulation or thrombolysis in addition to treatment of elevated intracranial tension.

The main line of treatment in CSVT is anticoagulation using heparin followed by oral anticoagulants; however, a lot of controversy occurs because of the risk of bleeding especially in the presence of hemorrhagic infarctions. Multiple randomized clinical trials were done to weigh the risk and benefits of anticoagulation in comparison to placebo. Three small trials were done in the beginning of the 21st century showing no significant benefit of anticoagulation in comparison to placebo [26-28]. However, around 10 years later, Coutinho et al. performed a meta-analysis including two randomized trials that showed absolute reduction in mortality by 13%, reduction in pulmonary embolism, and no increase in the risk of intracranial hemorrhage [29]. After stabilization of the acute phase, warfarin would be started and continued for three months as a total duration of treatment if the precipitating factor was transient, and for 6-12 months in the case of high risk of recurrence as in thrombophilia. The INR range should be between 2 and 3 [5].

Local thrombolysis and mechanical thrombectomy are sometimes used to restore the blood flow of thrombosed sinuses if the patient is clinically deteriorating with no adequate response to anticoagulation therapy [30].

Treatment of elevated intracranial pressure is of great importance in cases of CSVT. It usually includes mannitol infusion, surgical removal of hematomas, and surgical decompression hemi-craniectomy as a last resort. Lumbar punctures are also considered for relieving of intracranial pressure as long as space-occupying lesions are excluded [31,32].

## Conclusions

CSVT is a rare condition that predominantly affects young adults and children with female predilection resulting in venous congestion, brain edema, and venous infarctions. CSVT has multiple etiologies and highly variable clinical manifestations that need high clinical suspicion to be diagnosed. With the presence of effective medications and advanced neuroimaging, CSVT has become easier to diagnose, and has a favorable prognosis with good neurological outcomes. More research is needed to detect the effectiveness of other oral anticoagulants in the CSVT course of treatment.
